# Meeting of the Board of Officers and Directors

**Published:** 1883-01

**Authors:** A. H. Briggs

**Affiliations:** Secretary; Office of Dr. Geo. N. Burwell, No. 130 Pearl St.


MEETING OF THE BOARD OF OFFICERS AND DIRECTORS.

OFFICE OF DR. GEO. N. BURWELL,
No. 130 Pearl St., Dec. 15, 1882.

Dr. George N. Burwell, President of the Association, in the Chair.

All the members of the Board were present.

On motion of Dr. Hopkins, the President, Vice-President and Librarian of the Association were appointed a committee to draft by-laws, to be submitted at the next meeting of the Board.

On motion of Dr. Mann, the President appointed Drs. Granger, Hopkins and Frederick a committee to select a location for the library, and report at the next meeting of the Board.

Adjourned subject to the call of the President.

A. H. BRIGGS, *Secretary.*

